# Urodynamic effects of oxybutynin and tolterodine in conscious and anesthetized rats under different cystometrographic conditions

**DOI:** 10.1186/1471-2210-5-14

**Published:** 2005-10-11

**Authors:** Patrizia Angelico, Cristina Velasco, Luciano Guarneri, Giorgio Sironi, Amedeo Leonardi, Rodolfo Testa

**Affiliations:** 1 Pharmaceutical R & D Division – Recordati S.p.A. – Via Civitali 1, 20148 Milano, Italy

## Abstract

**Background:**

Antimuscarinic agents are the most popular treatment for overactive bladder and their efficacy in man is well documented, producing decreased urinary frequency and an increase in bladder capacity. During cystometry in rats, however, the main effect reported after acute treatment with antimuscarinics is a decrease in peak micturition pressure together with little or no effect on bladder capacity. In the present experiments we studied the effects, in rats, of the two most widely used antimuscarinic drugs, namely oxybutynin and tolterodine, utilising several different cystometrographic conditions. The aim was to determine the experimental conditions required to reproduce the clinical pharmacological effects of antimuscarinic agents, as seen in humans, in particular their ability to increase bladder capacity.

**Results:**

Intravenous or oral administration of tolterodine or oxybutynin in conscious rats utilized 1 day after catheter implantation and with saline infusion at constant rate of 0.1 ml/min, gave a dose-dependent decrease of micturition pressure (MP) with no significant change in bladder volume capacity (BVC). When the saline infusion rate into the bladder was decreased to 0.025 ml/min, the effect of oral oxybutynin was similar to that obtained with the higher infusion rate. Also, experiments were performed in rats in which bladders were infused with suramin (3 and 10 μM) in order to block the non-adrenergic, non-cholinergic component of bladder contraction. Under these conditions, oral administration of oxybutynin significantly reduced MP (as observed previously), but again BVC was not significantly changed.

In conscious rats with bladders infused with diluted acetic acid, both tolterodine and oxybutynin administered at the same doses as in animals infused with saline, reduced MP, although the reduction appeared less marked, with no effect on BVC.

In conscious rats utilized 5 days after catheter implantation, a situation where inflammation due to surgery is reduced, the effect of tolterodine (i.v.) and oxybutynin (p.o.) on MP was smaller and similar, respectively, to that observed in rats utilized 1 day after catheter implantation, but the increase of BVC was not statistically significant.

In anesthetized rats, i.v. administration of oxybutynin again induced a significant decrease in MP, although it was of questionable relevance. Both BVC and threshold pressure were not significantly reduced. The number and amplitude of high frequency oscillations in MP were unmodified by treatment.

Finally, in conscious obstructed rats, intravenous oxybutynin did not modify frequency and amplitude of non-voiding contractions or bladder capacity and micturition volume.

**Conclusion:**

Despite the different experimental conditions used, the only effect on cystometrographic parameters of oxybutynin and tolterodine in anesthetized and conscious rats was a decrease in MP, whereas BVC was hardly and non-significantly affected. Therefore, it is difficult to reproduce in rats the cystometrographic increase in BVC as observed in humans after chronic administration of antimuscarinic agents, whereas the acute effects seem more similar.

## Background

Overactive bladder is a chronic clinical syndrome characterized by urgency and frequency with or without urinary incontinence affecting millions of people worldwide [1,2]. Overactive bladder arises from uncontrolled contraction of the detrusor muscle during bladder filling [3]. Although what element(s) trigger(s) unstable contraction is not resolved; myogenic [4] or neural [5] theories have been suggested in the attempt to clarify the etiology of this bladder dysfunction.

Nevertheless, since contraction of the detrusor muscle and bladder emptying are primarily mediated by stimulation of muscarinic receptors by acetylcholine, anticholinergic agents are currently recommended as a first-line therapy for overactive bladder. Of the available antimuscarinic agents, oxybutynin and tolterodine are the most widely used to treat this condition [6,7].

As demonstrated by several investigations in patients, oxybutynin decreases urinary frequency, urgency and episodes of urge incontinence, in addition to increasing bladder volume at first desire to void, enhancing maximum bladder capacity and reducing maximum detrusor pressure during filling [6,8]. Similar results have been reported after administration of tolterodine in patients affected by overactive bladder [[6], and references therein].

Despite the favourable results observed in the clinical studies reported above with regard to the cystometrographic modifications induced by treatment with oxybutynin and tolterodine, the only effect generally observed in rats is a decrease in the maximum detrusor pressure at micturition [9-18].

Although purinergic mechanisms appear not to be involved to any extent in the normal function of human bladder [19], they are involved in human pathological conditions [19], and they are well documented for contraction of rat urinary bladder [20].

The aim of the present experiments was, therefore, to study the effect of oxybutynin and tolterodine in rats, using different cystometrographic conditions, attempting to find suitable experimental conditions able to reproduce the effects observed in humans, in particular the increase of bladder capacity.

A preliminary account of this work was presented in an abstract [21].

## Results

Tolterodine and oxybutynin were evaluated as the most widely utilized antimuscarinics.

In general, 2–3 scaled doses of each compound were administered. In some pilot experiments each dose of compound was tested with a matched control group treated with vehicle. In other experiments, control rats and animals treated with different doses of test compound were evaluated simultaneously.

### Effect of intravenous and oral administration in conscious rats utilized 1 day after catheter implantation

**Figure 1:**
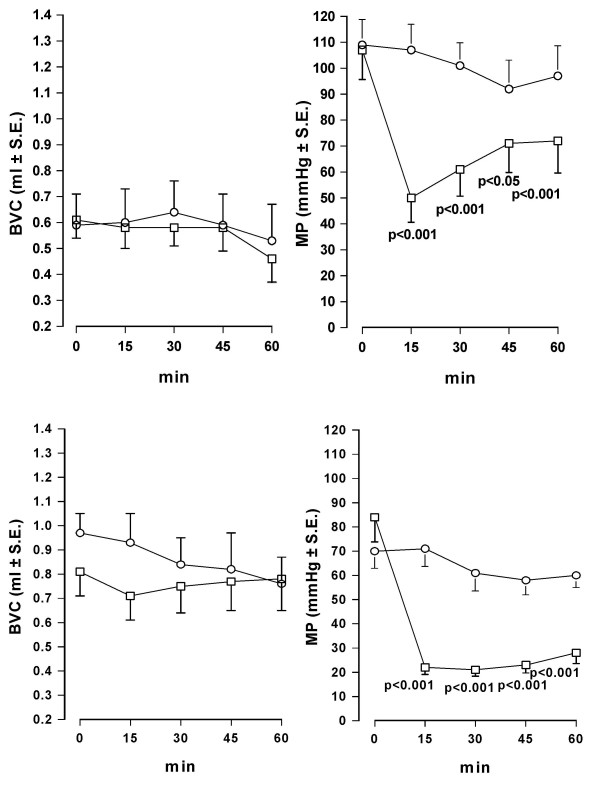
**Time-course of the effect of i.v. administration of oxybutynin and tolterodine on BVC and MP in conscious rats**. Data represent the mean (± S.E.) of BVC (ml) and MP (mmHg) values recorded before treatment (0 min) and at different times after administration of: upper panel – vehicle (0.1 ml/kg; circles; n = 6) or oxybutynin (0.3 mg/kg; squares; n = 8); lower panel – vehicle (0.1 ml/kg; circles; n = 7) or tolterodine (0.3 mg/kg; squares n = 8). Rats were utilized 1 day after catheter implantation. Statistical analysis indicates significativity vs vehicle-treated group at considered times.

**Figure 2:**
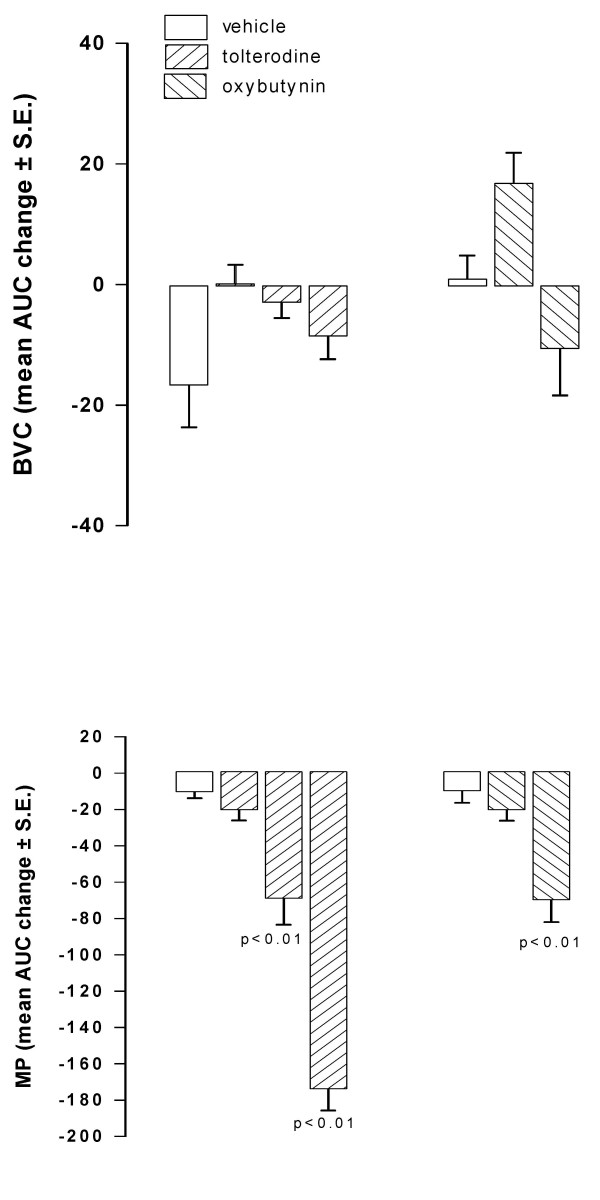
**Effect of i.v. administration of tolterodine and oxybutynin on BVC and MP in conscious rats**. Data represent the mean (± S.E.) AUC change of BVC and MP calculated as reported in the Method Section. Tolterodine was administered at 0.03 (n = 8), 0.1 (n = 9) and 0.3 (n = 8) mg/kg; oxybutynin at 0.1 (n = 5) and 0.3 (n = 8) mg/kg. Corresponding open bars represent changes recorded in the vehicle groups (n = 7). Rats were utilized 1 day after catheter implantation. Statistical significance was evaluated by ANOVA (and Dunnett's test).

**Figure 3:**
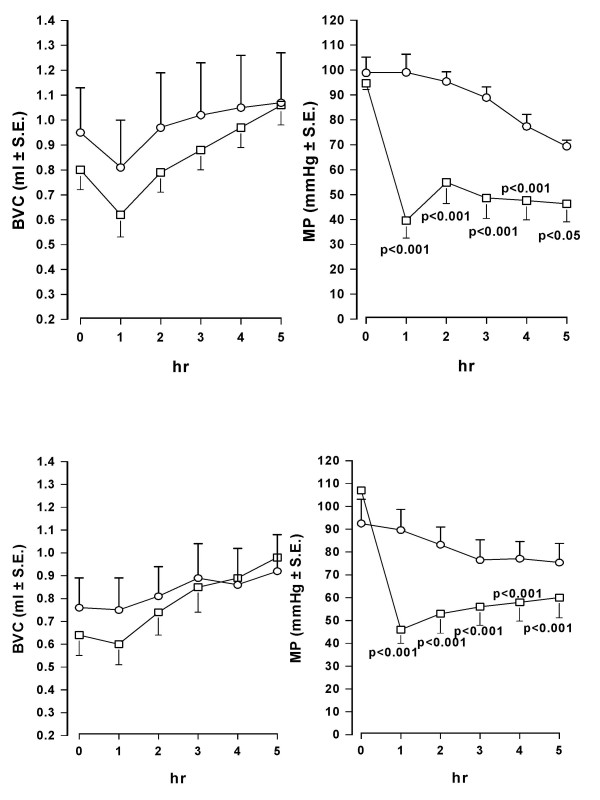
**Time-course of the effect of oral administration of oxybutynin and tolterodine on BVC and MP in conscious rats**. Data represent the mean (± S.E.) of BVC (ml) and MP (mmHg) values recorded before treatment (0 min) and at different times after administration of: upper panel – vehicle (2 ml/kg; circles; n = 7) or oxybutynin (3 mg/kg; squares; n = 7); lower panel – vehicle (2 ml/kg; circles; n = 7) or tolterodine (10 mg/kg; squares n = 8). Rats were utilized 1 day after catheter implantation. Statistical analysis indicates significativity vs vehicle-treated group at considered times.

**Figure 4:**
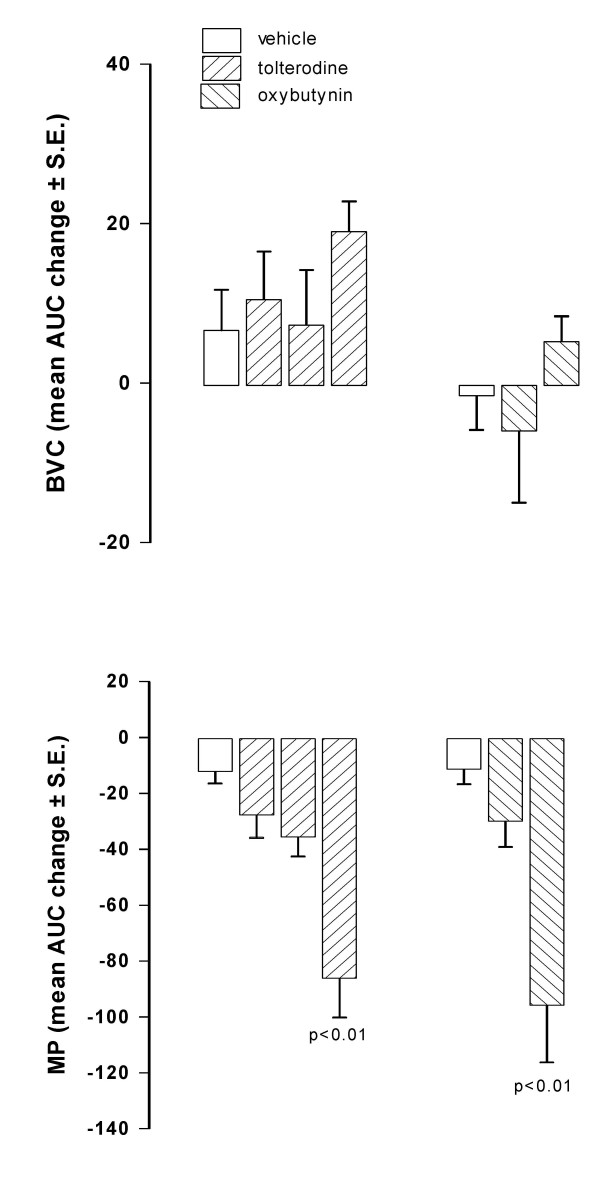
**Effect of oral administration of tolterodine and oxybutynin on BVC and MP in conscious rats**. Data represent the mean (± S.E.) AUC change of BVC and MP calculated as reported in the Method Section. Tolterodine was administered at 1 (n = 6), 3 (n = 7) and 10 (n = 8) mg/kg; oxybutynin at 1 (n = 7) and 3 (n = 7) mg/kg. Corresponding open bars represent changes recorded in the vehicle groups (n = 7). Rats were utilized 1 day after catheter implantation. Statistical significance was evaluated by ANOVA (and Dunnett's test).

When i.v. injected in conscious rats, oxybutynin (0.3 mg/kg) induced a prompt and marked reduction of MP showing a peak 15 min after injection. BVC, in contrast, was not modified in comparison with the values observed in control animals. Similar results were obtained after administration of the same dose of tolterodine (Fig. 1). After different doses of both antimuscarinics administration, a dose-dependent reduction of MP was observed, with no significant changes of BVC, tolterodine being substantially more potent than oxybutynin (Fig. 2). After oral administration of oxybutynin (3 mg/kg) and tolterodine (10 mg/kg), the time-course of BVC and MP values in control and treated animals was similar to that observed after i.v. administration (Fig. 3). The compounds induced a marked and statistically significant fall of MP reaching a peak 1 hr after administration with no significant effects on BVC. Dose-response curves obtained after administration of the antimuscarinics are shown in Fig. 4. Oxybutynin and tolterodine dose-dependently decreased MP.

**Figure 5:**
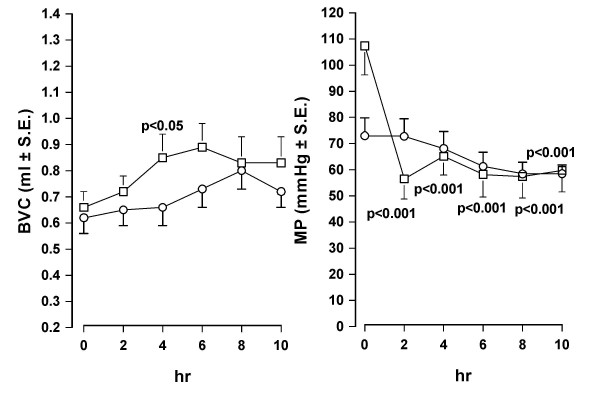
**Time-course of the effect of oral administration of oxybutynin on BVC and MP in conscious freely-moving rats under discontinuous cystometry**. Data represent the mean (± S.E.) of BVC (ml) and MP (mmHg) values recorded before treatment (0 min) and at different times after administration of vehicle (2 ml/kg; circles; n = 11) or oxybutynin (3 mg/kg; squares; n = 11).

The effects of oral administration of oxybutynin (3 mg/kg) were also evaluated in freely-moving rats using discontinuous cystometry, increasing observation period up to 10 hr after treatment. Discontinuous cystometry allows parameters to be recorded for extended periods of time, avoiding the detrimental effects on the levels of basal physiological urodynamic parameters seen in restrained rats following continuous bladder infusion for a long time (for example decrease in peak micturition pressure). Oxybutynin induced a decrease of MP similar to that observed previously during the first 2–4 hr after treatment (see Fig. 5 and the corresponding Fig. 3). The difference between treated and control group resulted statistically significant in spite of the figure course because it should be considered that it was evaluated on Δ values, as reported in the Method section. BVC values resulted significantly (p < 0.05) different in comparison with vehicle treated animals at 4 hr after administration. This result, in contrast to that previously obtained during classical cystometry, however, can be related to the behaviour of the control group, whose BVC values were extremely constant during the period.

**Figure 6:**
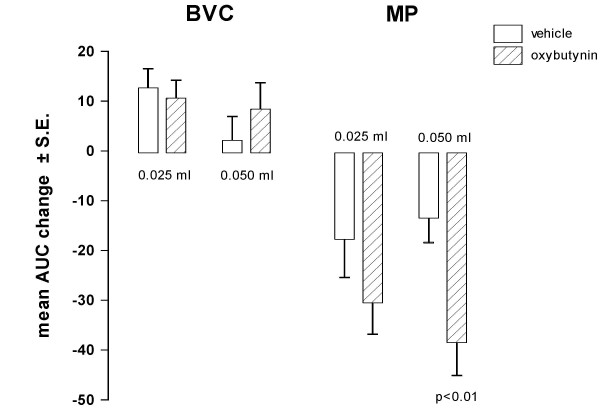
**Effect of oral administration of oxybutynin on BVC and MP in conscious rats under cystometry at different infusion rates**. Data represent the mean (± S.E.) AUC change of BVC and MP calculated as reported in the Method Section. Oxybutynin was administered at 1 mg/kg (n = 9). Corresponding open bars represent changes recorded in the vehicle groups (n = 8). Infusion rates utilized were 0.025 and 0.05 ml/min. Rats were utilized 1 day after catheter implantation. Statistical significance was evaluated by Student's t test.

To further study the effect of oxybutynin on BVC, the less inhibitory dose of oxybutynin on MP (oral dose of 1 mg/kg) was chosen and the saline infusion rate into the bladder was decreased from 0.1 ml/min to 0.05 and 0.025 ml/min. However, the effect of oxybutynin on BVC and MP was found to be similar to that obtained with the higher flow rate (Fig 6).

**Figure 7:**
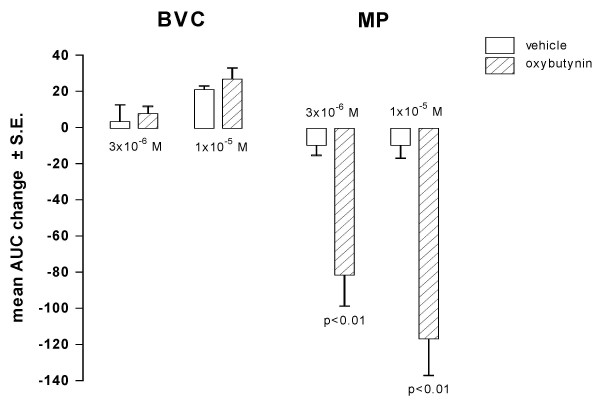
**Effect of oral administration of oxybutynin on BVC and MP in conscious rats with bladder infused with different suramin concentrations**. Data represent the mean (± S.E.) AUC change of BVC and MP calculated as reported in the Method Section. Oxybutynin was administered at 3 mg/kg (n = 8). Corresponding open bars represent changes recorded in the vehicle groups (n = 8). Suramin concentrations were 3 × 10^-6 ^and 1 × 10^-5 ^M. Rats were utilized 1 day after catheter implantation. Statistical significance was evaluated by Student's t test.

In order to block the non-adrenergic, non-cholinergic component of the contraction, experiments were performed in rats with bladders infused with suramin (3 and 10 μM). Suramin (10 μM) in vehicle-treated rats induced a significant increase (in comparison with basal values) in BVC (p < 0.01). Under these conditions, oral administration of 3 mg/kg of oxybutynin reduced significantly MP (as previously observed), but BVC was again not significantly increased (Fig 7). In normal unanesthetized rats, Igawa et al. [20] found that co-administration of atropine in rats with purinergic receptors desensitised by administration of α,β-methylene ATP induced urinary retention. In our experimental conditions, we did not observed overflow incontinence in rats with bladder infused with suramin and treated with oxybutynin, probably owing to the fact that suramin is not a specific antagonist [19] and, therefore, not all the P2X receptors were inhibited.

### Effect of intravenous and oral administration in conscious rats treated 5 days after catheter implantation

**Figure 8:**
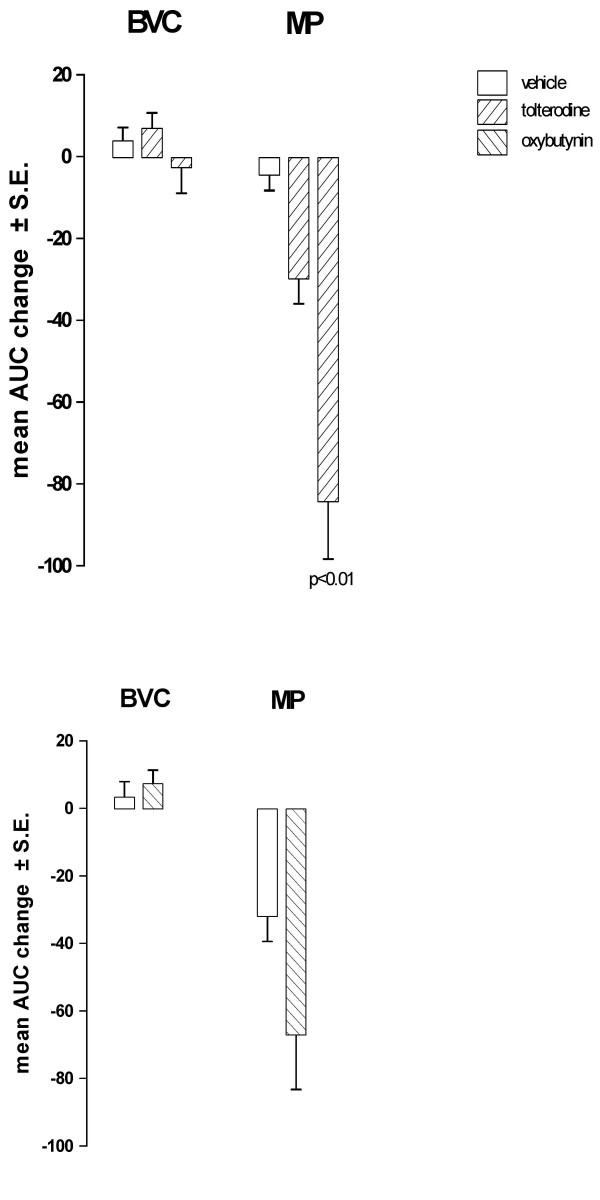
**Effect of tolterodine and oxybutynin on BVC and MP in conscious rats utilized 5 days after catheter implantation**. Data represent the mean (± S.E.) AUC change of BVC and MP calculated as reported in the Method Section. Tolterodine was i.v. administered at 0.1 (n = 7) and 0.3 (n = 7) mg/kg (upper panel). Oxybutynin was administered at 1 mg/kg (n = 7; lower panel). Corresponding open bars represent changes recorded in the vehicle groups (n = 7). Statistical significance was evaluated by ANOVA (and Dunnett's test).

Catheterization of the bladder induces inflammation that reaches a peak between 1–3 days after surgery. At 5 days post catheterization, inflammation is considerably lower than at 1 day after catheter implantation, as confirmed by the higher values of BVC recorded (about 1.3 – 1.7 ml, data not shown) than those at 1 day after surgery (see basal values in Fig 1 and 3). Under these conditions, i.v. administration of tolterodine dose-dependently decreased MP without affecting BVC (Fig. 8). The effect of tolterodine on MP was less than that observed in rats 1 day after surgery. The effect of oral administration of oxybutynin (1 mg/kg) on BVC and MP was not substantially different from that observed in rats utilized 1 day after catheter implantation.

### Effect of intravenous administration in anesthetized rats

Cystometrographic and electromyographic recordings performed during micturition in rats under anesthesia show that the striated muscle of external urethral sphincter (EUS) exhibits high-frequency bursting that induces corresponding high-frequency oscillations (HFO) in the pressure recorded intravescically. HFO are correlated with the bursting pattern in the EUS, and represent alternate contractions and relaxations of the urethral outlet, functioning like a pump to enhance urine flow. Consequently, pharmacological manipulations that may result in impaired EUS function might result in micturition disturbances, e.g. bladder-urethra dyssynergia.

In anesthetized rats, the i.v. administration of 0.3 mg/kg of oxybutynin (a dose inducing in conscious rats a significant decrease of MP) induced again a significant, although not so relevant decrease of MP, probably owing to the lower basal value of this parameter due to the anesthesia. Neither BVC nor threshold pressure showed a significant reduction. The number and amplitude of HFO were not modified by oxybutynin.

### Effect of intravenous administration in conscious rats with bladder infused with diluted acetic acid

**Figure 9:**
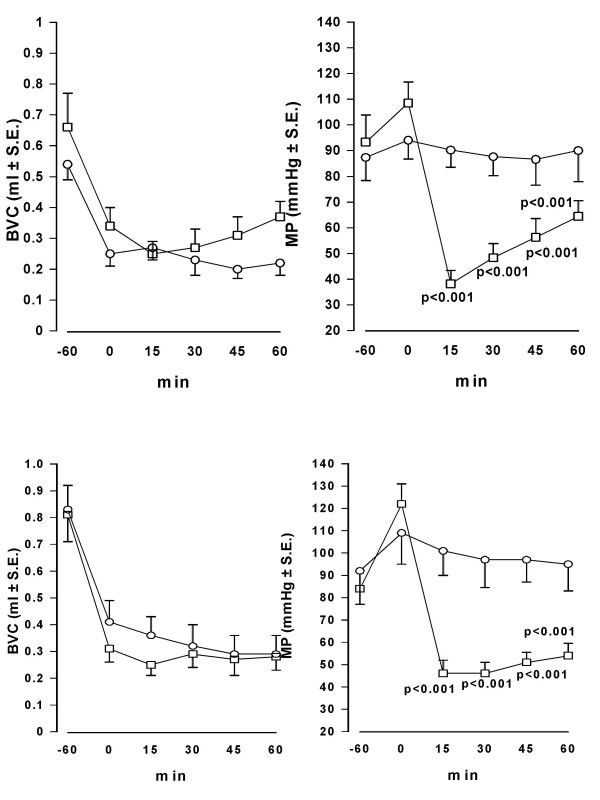
**Time-course of the effect of i.v. administration of oxybutynin and tolterodine on BVC and MP in conscious rats with bladder infused with diluted acetic acid**. Data represent the mean (± S.E.) of BVC (ml) and MP (mmHg) values recorded before acetic acid infusion (-60 min), at the end of the first hr of acid infusion (0 min) and at different times after administration of: upper panel – vehicle (0.1 ml/kg; circles; n = 8) or oxybutynin (1 mg/kg; squares; n = 8); lower panel – vehicle (0.1 ml/kg; circles; n = 7) or tolterodine (0.3 mg/kg; squares n = 8). Rats were utilized 1 day after catheter implantation and during continuous infusion of acid. Statistical analysis indicates significativity vs vehicle-treated group at considered times.

Bladders of conscious rats catheterized from 1 day were infused for a control period of 60 min with saline. Then the infusion was switched to 0.2% acetic acid. At the end of the first hour of bladder infusion with the irritant, BVC was markedly and significantly reduced (about 40 – 60%; p < 0.01) in all groups of animals, indicating bladder hyperactivity due to pain and an increase in afferent nerve firing. Micturition pressure was less affected and generally increased (Fig. 9).

In the groups of animals treated with vehicle, the continuous infusion of the bladder with acetic acid during the second hour of experiment induced a further decrease of bladder capacity (generally 10–30%), although this further reduction was not statistically significant. MP did not change during the period. Oxybutynin (1 mg/kg i.v.) and tolterodine (0.3 mg/kg i.v.) had no effect on BVC, but caused a rapid and marked decrease of MP similar to that observed in rats during saline infusion of the bladder (Fig. 9).

**Figure 10:**
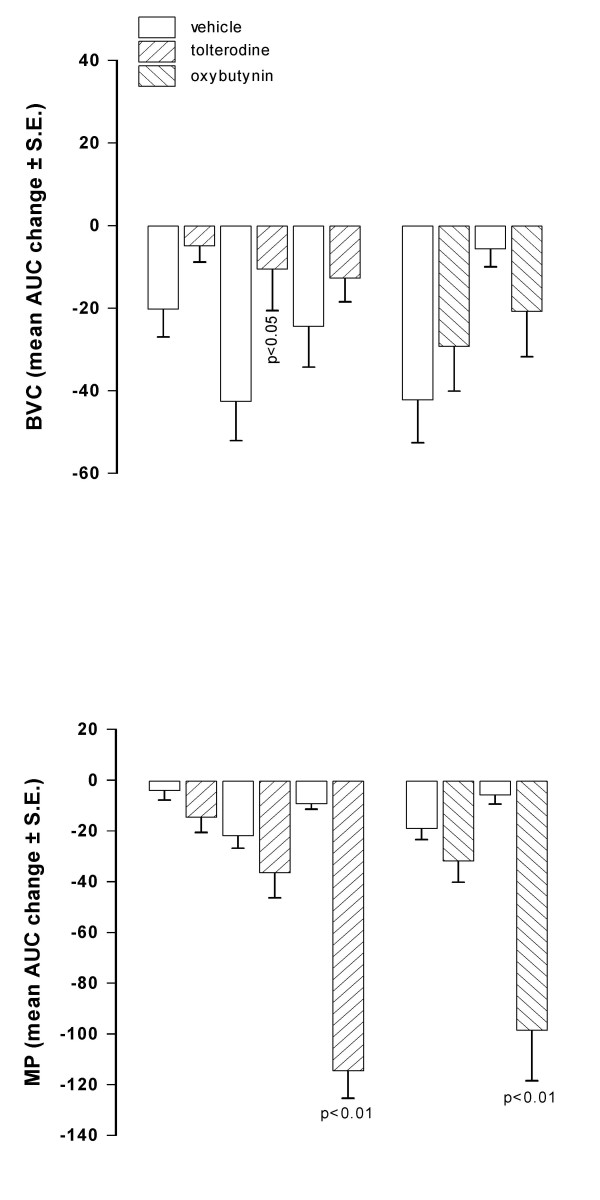
**Effect of i.v. administration of tolterodine and oxybutynin on BVC and MP in conscious rats with bladder infused with diluted acetic acid**. Data represent the mean (± S.E.) AUC change of BVC and MP calculated as reported in the Method Section (basal values were those recorded at the end of the first hr of acid infusion). Tolterodine was administered at 0.03 (n = 7), 0.1 (n = 6) and 0.3 (n = 8) mg/kg; oxybutynin at 0.1 (n = 8) and 0.3 (n = 8) mg/kg. Corresponding open bars represent changes recorded in the vehicle groups (n = 7). Rats were utilized 1 day after catheter implantation. Statistical significance was evaluated by ANOVA (and Dunnett's test) or Student's t test.

After the administration of the same i.v. doses as utilized in normal, saline-infused rats, both tolterodine and oxybutynin reduced MP, although the reduction appeared less marked (Fig. 10). The antimuscarinics generally showed only a trend to reverse the decrease in BVC observed during the second hr of acid infusion. However, the intermediate dose of tolterodine produced a significant difference (p < 0.05) between control and treated rats with regard to BVC.

### Effect of intravenous administration in conscious obstructed rats

In rats, bladder hypertrophy secondary to bladder outlet obstruction induces bladder instability characterized by the presence of non-voiding contractions (NVC) during filling. Repeated cystometry in control obstructed rats injected with saline gave reproducible results in terms of frequency and amplitude of spontaneous contractile activity (NVC). BVC and MV in these animals were also not significantly changed. Oxybutynin (0.3 mg/kg i.v.) gave results similar to control animals (Table 2).

**Table 2:** Effect of intravenous administration of oxybutynin on cystometrographic parameters in conscious obstructed rats

	BVC (ml)	MV (ml)	NVC: frequency (N°/2 min)	NVC: amplitude (mmHg)
Controls				
Before	1.27 ± 0.16	1.36 ± 0.24	5.3 ± 0.9	5.2 ± 0.9
After	1.14 ± 0.25	1.32 ± 0.30	4.9 ± 0.9	4.9 ± 0.9

Oxybutynin 0.3 mg/kg				
Before	1.98 ± 0.12	2.02 ± 0.46	5.9 ± 0.4	11.7 ± 1.8
After	2.58 ± 0.70	2.77 ± 0.50	5.5 ± 0.6	9.7 ± 2.1

Data are expressed as mean ± s.e. mean. BVC: bladder volume capacity; MV: micturition volume; NVC: non-voiding contractions. * = p < 0.05; ** = p < 0.01 before vs after administration (Student's t test).

## Discussion

A summary of the different models and conditions utilized to evaluate the effects of tolterodine and/or oxybutynin is shown in Table 3.

**Table 3:** Summary of results obtained after treatment with tolterodine and/or oxybutynin in the different models/conditions utilized.

**Models/conditions**	**Effect on BVC**	**Effect on MP**
Conscious rats – 1 day after surgery – i.v. and p.o. administration	=	↓↓
Conscious (freely-moving/discontinuos cystometry) rats – 1 day after surgery – p.o. administration	=	↓
Conscious (cystometry at different rate of filling) rats – 1 day after surgery – p.o. administration	=	↓
Conscious (bladder infused with suramin) rats – 1 day after surgery – p.o. administration	=	↓
Conscious (bladder infused with acetic acid) rats – 1 day after surgery – p.o. administration	= (↑)	↓↓
Conscious (obstructed) rats – 2 days after surgery – i.v. administration	=	n.e.
Conscious rats – 5 days after surgery – i.v. and p.o. administration	=	↓
Anesthetized rats – at the day of surgery – i.v. administration	=	↓

= not significant change; ↓: decrease; ↓↓: dose-dependent decrease; n.e. = not evaluated (since the ligature around the urethra was not removed before cystometry, peak micturition pressure was not considered in these experiments); = (↑): some non dose-dependent effect was seen after tolterodine but not after oxybutynin.

Cystometrographic evaluation performed in conscious normal rats utilized one day after catheter implantation showed that neither oxybutynin nor tolterodine increased BVC after oral or i.v. administration. In agreement with previously reported data [9,13,15,16], however, both drugs induced a strong dose-dependent decrease of MP. Furthermore, this behaviour was maintained when a discontinuous cystometry was performed or when different filling rates were utilized.

Available evidence indicates that arachidonic acid metabolites produced along the cyclooxygenase pathway are involved in the physiological regulation of micturition during reflex activation of the urinary bladder. Furthermore, endogenous prostaglandins are produced locally following distension of the bladder wall and modulate the afferent branch of reflex micturition by lowering the threshold for eliciting voiding contractions, serving as a link between detrusor muscle stretch produced by bladder filling and activation of capsaicin-sensitive afferents [22-25]. Different authors [26,27] have shown that cystometrograms in conscious rats with bladders infused with saline and recorded during the first 1–3 days after catheter implantation showed bladder overactivity with relatively low urine volume and a high frequency of micturition. Cystitis is also present at this time and is characterized by edema in the submucosa and an increased tissue content of prostaglandins that stimulate capsaicin-sensitive sensory fibers in the afferent branch of the micturition reflex [27]. In the present study, cystometrographic recordings performed in conscious rats one day after catheter implantation and during saline infusion of bladder, confirmed that bladder capacity is significantly reduced in comparison with BVC values observed 5 days after catheter implantation, as previously reported [27]. It is therefore conceivable that in our experiments, performed under conditions of bladder inflammation, that a strong influence of prostaglandin levels on afferent firing was present. On the other hand, when the activity of oxybutynin or tolterodine was evaluated in rats 5 days after catheter implantation, the same effect as seen after 1 day, still occurred; namely, a decrease in MP with no change in BVC. These results indicate that the involvement of the inflammatory mediators is not the reason for the lack of antimuscarinic activity on BVC.

The involvement of ATP in non adrenergic, non cholinergic (atropine-resistant) contraction of urinary bladder is well documented [20]. Recently, it has also been demonstrated that ATP is released from the urothelium of isolated urinary bladder following increased intraluminal pressure [28]. Furthermore, it has been reported that intravesical ATP stimulates the micturition reflex in awake, freely moving rats [29] and that during cystometry the number of impulses generated in the afferent neurons was halved by treatment with suramin [19]. Consequently, suramin infusion into rat bladder has been reported to increase BVC [30]. Outflow obstruction may be associated with changes in the cholinergic function of the bladder associated with an increase of atropine-resistance. Again, the bladder instability seen in obstructed rats seems to be particularly related to the atropine-resistant contraction component, where ATP and prostaglandins play an important role [14]. However, we found that oxybutynin did not increase BVC either in rats under infusion of the bladder with suramin, or in obstructed rats. In addition, in this last experimental condition, oxybutynin did not modify the frequency and amplitude of the non-voiding contractions. These findings are in agreement with the lack of activity shown by tolterodine in the same model [31], whereas Kwak and Lee [14] reported a significant decrease of frequency and amplitude of the non voiding contractions in anesthetized rats after intraarterial administration of 1 mg/kg oxybutynin.

Although the mechanism of action of antimuscarinic agents used for the treatment of overactive bladder (such as oxybutynin and tolterodine) is thought to be due mainly to suppression of detrusor contraction through blockade of M3 muscarinic receptors on detrusor smooth muscle, an effect on central muscarinic receptors, located in the brain cannot be ruled out as both compounds pass into the central nervous system [32].

Effects on the lower urinary tract of drugs acting on central nervous system muscarinic receptors have been reported by several investigators [17,33-35] Following i.c.v. or i.t. administration in conscious normal rats of oxotremorine methiodide, a muscarinic agonist, a dose-dependent increase of BVC was observed. The muscarinic antagonist atropine did not change BVC after i.t. administration, and increased BVC after i.c.v. administration only in very high doses [36]. Both oxybutynin and tolterodine i.c.v. administered showed no (oxybutynin) or little (tolterodine) augmenting effect on BVC [17]. On the other hand, several Authors reported that oral oxybutynin administered in conscious rats with lesions at the basal forebrain [11,18], increases BVC. These findings seem to indicate that the inhibitory muscarinic mechanisms that can be activated by exogenously administered agonists seem to be inactive under normal conditions [36], but are working in lesioned animals. These considerations, therefore, can explain the lack of central activity on BVC of antimuscarinics tested in conscious normal rats.

Muscarinic receptors are also found on bladder urothelial cells, where their density may be even higher than in detrusor muscle. Evidence has been reported for a release of acetylcholine from urothelium and/or nerves during bladder filling, and acetylcholine may act directly on afferent nerves to initiate the micturition reflex or to enhance the myogenic contractile activity of the detrusor. If this is correct, blockade of muscarinic receptors should be expected to reduce bladder tone during storage and to increase bladder capacity. This effect is observed after treatment with antimuscarinics in normal individuals as well as in patients with detrusor overactivity [37]. In conscious rats, however, infravescical administration of different concentrations of oxybutynin did not increased BVC, but significantly decreased MP [12]. Furthermore, Kim et al. [38] recently showed that bladder infusion of different antimuscarinic agents (including oxybutynin) at concentrations equivalent to urine concentration in humans with oral application of these drugs, did not modified BVC when tested in normal rats but only inhibited bladder overactivity induced by intravesical instillation of carbachol.

## Conclusion

Despite the different experimental conditions utilized, the main effect of the antimuscarinics tested on cystometrographic parameters in anesthetized and conscious rats is a decrease of MP, whereas BVC is hardly and, generally, non-significantly affected, suggesting that the block of bladder muscarinic receptors is the only mechanism that can be affected and evaluated after treatment with these compounds in this animal species.

Although urodynamic assessment showed significant comparable increases in bladder capacity following repeated treatment with oxybutynin and tolterodine in humans [6,7], the published papers reporting the acute effect of these antimuscarinics in man are confusing, since oxybutynin seems devoid of effect [39], whereas tolterodine increased the volumes at which subjects experienced the first sensation of bladder filling and normal desire to void [40]. It seems therefore difficult to reproduce in rats the cystometrographic effects observed in humans after chronic administration of these compounds. The effects observed in rats after acute administration (although obtained at doses about 10–100-fold higher than those used in ref. 39 and 40) seem more similar to those recorded in patients treated with a single dose, with the exception of the modification in symptoms that can not be recorded in animals.

## Methods

The effects of the tested compounds on rats urodynamic parameters were evaluated by cystometrographic models in conscious and anesthetized animals. In anesthetized or obstructed animals (i.v. administration only) cystometry was performed on female rats (250–350 g b.w.). Conscious male rats (300–400 g b.w.) were used to evaluate the effect of tested compounds both after i.v. and oral administration.

Animals were housed with free access to food and water and maintained on a forced 12 hr light-dark cycle at 20–24°C for at least one week before the experiments were carried out. The animals were handled according to internationally accepted principles for the care and welfare of laboratory animals (E.E.C. Council Directive 86/609, O. J. no L358, 18/12/86).

### Surgical procedures

To obtain bladder outlet obstruction, female rats were anesthetized with intraperitoneal administration of 3 ml/kg of equithensin solution (pentobarbital g 1.215, chloral hydrate g 5.312, magnesium sulphate g 2.657, ethanol ml 12.5, propylene glycol ml 49.5, distilled water to 125 ml of final volume) and then the bladder and proximal urethra were exposed via a lower abdominal midline incision. A silk ligature was placed around the urethra and tied in the presence of an intraluminally placed indwelling polyethylene cannula with an outside diameter of 1.22 mm. After removing the polyethylene cannula, the abdominal wall was sutured and then antibiotic medication (penicillin G 200,000 I.U./kg and streptomycin 300,000 I.U./kg i.m.) was administered. Obstructed rats were utilized for cystometry 3 weeks after urethral ligature.

To insert the catheter into the bladder, female rats were anesthetized with subcutaneous injection of urethane 1.25 g/kg (5 ml/kg). Animals were then placed in a supine position and an approximately 10 mm midline incision was made in the shaved and cleaned abdominal wall. The urinary bladder was gently freed from adhering tissues, emptied and then cannulated, via an incision at the dome, with a polyethylene cannula (ID 0.58 mm, OD 0.96 mm), which was permanently sutured with silk thread. For i.v. injection, another polyethylene cannula with the same characteristics and filled with heparine (40 UI/ml) in physiological saline was inserted into the jugular vein.

Obstructed female or male rats utilized 1 or 5 days after surgery were anesthetized with i.p. injection of equithensin (3 ml/kg) and catheters implantation was performed as above.

In female rats utilized upon anesthesia, the cannulae were exteriorized through a subcutaneous tunnel in the breast-bone area. In male and female rats awake utilized, the cannulae were exteriorized in the retroscapular area, where they were connected with a plastic adapter, in order to avoid the risk of removal by the animal. In male rats submitted to discontinuous cystometry, the free end of bladder cannula was connected to a swivel at the top of the cage, thus allowing free movements to animals in a 20 × 25 cm size cage. A rigid fluid-filled tubing connected to the swivel provided undistorted transmission of the bladder pressure to the transducer, which was placed outside the cage, at a height corresponding to the position of rat bladder.

### Cystometry procedures

The free tip of the bladder cannula was connected by a T-shape tube to a pressure transducer and to a peristaltic pump for a constant rate continuous infusion of saline solution (at room temperature) into the urinary bladder.

In anesthetized female normal rats, the urodynamic parameters were recorded continuously using a MacLab/8SP interface with Chart Software v. 4.1.2. (AD Instrument). In conscious rats, the urodynamic parameters were obtained from the cystometrogram recorded on a chart poligraph (Rectigraph SAN-EI 8K).

In anesthetized female rats the following parameters were evaluated: bladder volume capacity (BVC), defined as the volume (in ml) of saline infused into the bladder and necessary to induce detrusor contraction followed by micturition; micturition pressure (MP, in mm Hg) defined as the maximal intravesical pressure induced by contraction of the detrusor during micturition; threshold pressure (TP, in mmHg) i.e. the difference between intraluminar basal pressure and pressure value recorded just before micturition; the number (n.HFO) and the amplitude (a.HFO, in mmHg) of high-frequency oscillations recorded in 1 sec at the middle of the expulsion time phase.

In conscious female obstructed rats, the number and the mean amplitude of the spontaneous bladder contractions, present during bladder filling but without urine emission and termed "non-voiding contractions" (NVC), were evaluated for the 2 min time prior to micturition. In addition, BVC, MP and micturition volume (MV) were evaluated.

In conscious normal male rats, only BVC and MP values (defined as above) were obtained from the cystometrograms.

### Cystometric investigation in conscious normal rats

Rats were generally utilized one day after catheter implantation. Some experiments were carried out in rats utilized 5 days after surgery.

On the day of the experiment, conscious rats were placed in Bollman's cages and the recording of cystometrographic parameters started after a stabilization period of 20 min. Saline infusion into the bladder was generally at a constant rate of about 0.1 ml/min. Some experiments were performed decreasing the rate of saline infusion at 0.05 and 0.025 ml/min. Basal BVC and MP values were evaluated as the mean of two complete and reproducible cystometrograms during the pretreatment period (basal). Then the animals were treated intravenously or orally with the test compound (or vehicle) under continuous infusion of the bladder with saline, and changes in BVC and MP were evaluated for 60 or 300 min, respectively.

**Figure 11:**
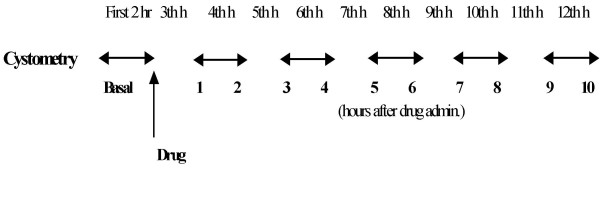
**Scheme of discontinous cystometry in conscious rats**. Bidirectional arrows indicate hourly periods of bladder infusion, vertical arrow indicates drug administration.

Discontinuous cystometry was performed for 12 hours (2 hours for basal recording and 10 hours after administration of drugs), through a discontinuous infusion of warm saline solution into the urinary bladder (1 h cycle) as reported in Fig. 11.

The effect of the following treatments was evaluated:

- i.v. administration of tolterodine (0.03 – 0.1 and 0.3 mg/kg) and oxybutynin (0.1 and 0.3 mg/kg) in conscious rats utilized 1 day after catheter implantation;

- p.o. administration of tolterodine (1 – 3 and 10 mg/kg) and oxybutynin (1 and 3 mg/kg) in conscious rats utilized 1 day after catheter implantation;

- p.o. administration of oxybutynin (3 mg/kg) in conscious freely-moving rats upon discontinuous cystometry;

- p.o. administration of oxybutynin (1 mg/kg) in conscious rats utilized 1 day after catheter implantation and with saline infusion rate of 0.025 and 0.05 ml/min;

- p.o. administration of oxybutynin (3 mg/kg) in conscious rats utilized 1 day after catheter implantation and with suramine infusion into the bladder at 3 × 10^-6 ^and 1 × 10^-5 ^M concentration.

- i.v. administration of tolterodine (0.1 and 0.3 mg/kg) in conscious rats utilized 5 days after catheter implantation;

- p.o. administration of oxybutynin (1 mg/kg) in conscious rats utilized 5 days after catheter implantation;

### Cystometric investigation in anesthetized normal rats

Rats were utilized on the day of catheter implantation. Saline infusion into the bladder was at a constant rate of about 0.1 ml/min. After stabilization, basal values of the considered parameters were evaluated as mean value from the second and third cystometrogram recorded after i.v. injection of vehicle. Then, the animals were treated with the compound tested (or again with vehicle for the control group) and changes induced by treatment were evaluated by considering the value of the cited parameters as mean of the second and third cystometrogram after treatment.

The effect of i.v. administration of oxybutynin 0.3 mg/kg was evaluated.

### Cystometric investigation in rats with bladder infused with diluted acetic acid

In conscious rats utilized 1 day after catheter implantation, saline solution was infused into bladder until stabilization of cystometrograms was achieved. At this point, bladder infusion was switched from saline to 0.2% acetic acid solution. Infusion rate was always 0.1 ml/min. One hr after, the rats were injected intravenously with the test compound (or vehicle) and changes in BVC and MP were evaluated for the following 60 min under continuous infusion of acetic acid. The effect of i.v. administration of tolterodine (0.03 – 0.1 and 0.3 mg/kg) and oxybutynin (0.1 and 0.3 mg/kg) was evaluated.

### Cystometric investigation in conscious obstructed rats

Rats were utilized two days after catheter implantation. Saline infusion into the bladder was at a constant rate of about 0.17 ml/min. From the cystometrograms of the obstructed rats, the number and the mean amplitude of the spontaneous bladder contractions, present during bladder filling without urine emission and termed "non-voiding contractions" (NVC) were evaluated for the 2 min time prior to micturition. In addition, BVC and micturition volume (MV) were evaluated. Values of the parameters reported above were expressed as mean values obtained from two similar cystometrograms recorded just before (basal values) and as mean of the second and third cystometrogram after treatment. Since the ligature around the urethra was not removed before cystometry, peak micturition pressure was not considered in these experiments.

The effect of i.v. administration of oxybutynin 0.3 mg/kg was evaluated.

### Data analysis

Data were always expressed as mean ± S.E. of the mean.

Statistical significance of the change of the different parameters recorded in anesthetized normal rats and conscious obstructed rats (before vs after treatment) was evaluated by Student's t test for paired data.

Statistical analysis of data involving time-course of BVC and MP values was performed by S.A.S./STAT software, version 6.12. The difference between vehicle and active treatments effect at different times was evaluated on values (for each rat the value at considered time minus basal value) of BVC and MP, using the general linear model procedure, repeated measures ANOVA, a univariate test of hypotheses for within subjects effects and ANOVA of contrast variables.

BVC and MP values of each rat were also transformed in AUC data. Statistical significance was evaluated by ANOVA (and Dunnett's test) or by Student's t test.

## Authors' contributions

PA, CV and GS carried out the experiments on the different animal models. LG partecipated in the design of the study and performed the statistical analysis. RT conceived of the study, partecipated in its design coordination and drafted the manuscript. AL conceived and performed the supervision of the study.

All authors read and approved the final manuscript.

**Table 1:** Effect of intravenous administration of oxybutynin on cystometrographic parameters in anesthetized rats

	BVC (ml)	TP (mmHg)	MP (mmHg)	HFO (number)	HFO (amplitude)
Controls					
Before	0.47 ± 0.04	4.74 ± 0.55	25.0 ± 3.6	4.2 ± 0.2	1.82 ± 0.19
After	0.47 ± 0.04	4.88 ± 0.58	20.9 ± 2.0	4.6 ± 0.2	1.63 ± 0.11

Oxybutynin 0.3 mg/kg					
Before	0.44 ± 0.11	3.44 ± 0.84	20.3 ± 2.3	4.6 ± 0.2	1.68 ± 0.27
After	0.27 ± 0.05	2.34 ± 0.51	15.5 ± 1.7 **	4.2 ± 0.7	1.63 ± 0.24

Data are expressed as mean ± s.e. mean. BVC: bladder volume capacity; TP: threshold pressure; MP: micturition pressure; HFO: high frequency oscillations (number and amplitude). * = p < 0.05; ** = p < 0.01 before vs after administration (Student's t test).
